# The Impact of Flipped Classroom Approach on Critical Thinking, Self‐Esteem, and Learning Retention of Nursing Undergraduate Students: A Cluster Randomized Study

**DOI:** 10.1002/hsr2.71271

**Published:** 2025-10-18

**Authors:** Mahsa Maryami, Mahboobeh Hosseinimoghadam, Khatereh Rostami

**Affiliations:** ^1^ Nursing and Midwifery School, Community Based Psychiatric Care Research Center Shiraz University of Medical Sciences Shiraz Iran; ^2^ Department of Nursing, School of Nursing and Midwifery Shiraz University of Medical Sciences Shiraz Iran

**Keywords:** critical thinking, final exam score, flipped classroom, nursing undergraduate students, self‐esteem

## Abstract

**Background and Aims:**

The flipped classroom model has emerged as a potentially transformative approach in nursing education, reversing conventional learning activities to prioritize active, student‐centered engagement. This study aimed to determine the impact of flipped classroom on critical thinking, self‐esteem, and learning retention of nursing undergraduate students.

**Methods:**

This experimental study used a cluster randomization approach, in which nursing students were assigned to either the intervention or control group based on their semester of enrollment. All sixth‐semester nursing students (42 students in the first semester and 45 students in the second semester) were included. The duration of the study was 1 year, with students entering the study in two semesters. Data were collected using the California Critical Thinking Skills Test B, Coopersmith Self‐Esteem Inventory, and two exam scores (final and 1‐month delayed retest). In the intervention group, students were taught using the flipped learning strategies for practical learning. Data were analyzed using SPSS v21.

**Results:**

Before the intervention, no significant difference was found between the groups with regard to critical thinking, self‐esteem, and learning retention. However, after the intervention, a significant difference was found between the groups in critical thinking (*p* < 0.001, *d* = 1.8), self‐esteem (*p* < 0.001, *d* = 1.2), and retention (*p* < 0.001, *d* = 0.9) compared to the control group (*p* > 0.05).

**Conclusion:**

The findings suggest that the flipped classroom approach positively influences critical thinking skills, self‐esteem, and learning final exam score among nursing undergraduate students. Instructors should prioritize the use of the flipped classroom method in competency‐based training courses (such as crisis management), and this approach should be reinforced by training faculty in digital technologies. Future research should also examine the long‐term effects of this method and its feasibility in different clinical settings.

## 1 Introduction

Considering the important role that nurses play in providing healthcare services and making clinical decisions, training nursing students with high skills to deliver safer and more appropriate care is essential [1, 2]. One of the essential skills that nursing students need to provide safe and effective care is critical thinking [3]. Critical thinking is a cognitive process that helps individuals evaluate and analyze information, enabling them to make the best decisions and achieve better outcomes [4]. Many studies have shown that the average critical thinking skills among nursing students are intermediate or low due to the current nursing school curriculum, which is not inherently designed to address students' critical thinking skills [5]. Lauvie‐Tremblay and colleagues showed that nursing students are often not confident in their work in the field, which hinders their ability to provide quality and effective care [6, 7]. One of the important dimensions of education for nursing students is the development of noncognitive skills such as self‐esteem in the university environment, as self‐esteem acts as a stress‐moderating factor and students with higher self‐esteem are able to make better clinical decisions in stressful situations [8]. Self‐esteem, a psychological construct linked to stress resilience and adaptive decision‐making, further influences clinical performance [9]. Yet, conventional lecture‐based education often fails to nurture self‐esteem, as passive learning formats prioritize rote memorization over reflective engagement [10]. This disconnect is exacerbated in competency‐based courses like crisis management, where high‐stakes decision‐making demands both cognitive mastery and psychological readiness [11, 12].

In most cases, students' lack of mastery over educational materials leads to a decrease in self‐esteem and also prevents the full blossoming of their talents and abilities in providing patient care [13]. One of the ways to evaluate it is by measuring the extent of memorization and retention of previously learned material [14]. By “recall,” we mean the ability to retain, store, and retrieve past experiences. Memory occurs when learning has already taken place, and when needed, part of the short‐term memory information connects with previous information and is transferred to long‐term memory [15].

Most nursing education programs use conventional teaching methods in which nursing instructors act as transmitters of knowledge to students. The limitation of these transferable teaching models is that learners are not actively involved in processing information. In conventional education, professors present large amounts of course information through lectures, while students are busy taking notes and do not have the opportunity to connect new educational content with their previous experiences and perceptions. In this method, the process of critical thinking is overlooked, and consequently, the knowledge gained will be forgotten shortly after the exam [16]. The flipped classroom is an innovative approach for transforming medical and nursing education. It is a learner‐centered educational method that reverses conventional in‐class and out‐of‐class learning activities. In this method, instead of teaching in an academic environment, lessons are learned at home and learning of educational materials is done outside the classroom and individually. After that, troubleshooting and homework are done in an interactive and dynamic environment, in the presence of professors [17, 18]. In such a way that students study educational lectures through slides or educational videos before class and engage in problem‐solving, discussion, and debate about the learned material during class [19]. Zulhamdi and colleagues showed that medical students can achieve independent learning through the flipped classroom and enhance their skills in patient care by fostering critical thinking [5]. While some studies report its success in fostering critical thinking and retention [14, 15], others reveal mixed outcomes. For instance, Hill et al. [20] found no significant improvement in self‐esteem among nursing students despite gains in knowledge retention, suggesting a disconnect between cognitive and affective outcomes [20].

This study is anchored in Constructivist Learning Theory (CLT), which posits that learners actively construct knowledge through social interaction, reflection, and problem‐solving [17]. CLT aligns with the flipped classroom's emphasis on preclass preparation (self‐directed learning) and in‐class collaboration (peer discourse), both critical for bridging cognitive and affective domains [18].

By framing the flipped classroom within CLT, this study addresses a key research gap:

How do constructivist principles mediate the impact of flipped learning on critical thinking, self‐esteem, and learning retention in nursing education?

### 1.1 The Aim of the Research

Determining the impact of the flipped classroom on critical thinking, self‐esteem, and learning retention in nursing undergraduate students.

## 2 Methods

### 2.1 Population and Sample

This cluster randomized study was conducted at Shiraz University of Medical Sciences in Iran during the 2022–2023 academic year. The study population comprised sixth‐semester undergraduate nursing students enrolled in the Crisis Management course. The intervention group consisted of 45 students enrolled in the second semester, while the control group included 42 students from the first semester.

Sample size: the total sample size (*N* = 87) was determined based on similar interventional studies in nursing education [21], which typically require 40–50 participants/group to detect moderate effect sizes with 80% power and a 0.05 significance level.

### 2.2 Study Setting

The setting for this study was the Hazrat Fatemeh Nursing and Midwifery School in Shiraz.

### 2.3 Inclusion Criteria

- Enrollment as a sixth‐semester undergraduate nursing student at Shiraz University of Medical Sciences.
- Participation in the Crisis Management course during the study period.
- Willingness to participate in the study and provide informed consent.

### 2.4 Exclusion Criteria

- Absence from more than one session of the course.
- Failure to complete either the immediate postcourse exam or the 1‐month delayed exam.
- Withdrawal from the study at any point.

### 2.5 Sampling Method

A cluster randomization design was employed in this experimental study. Students were not individually randomized; instead, all sixth‐semester nursing students enrolled in the first academic semester were assigned to the control group (*n* = 42), and those enrolled in the following semester were assigned to the intervention group (*n* = 45). Participants were recruited through the university's course registration system. All eligible students enrolled in the Crisis Management course during the specified semesters were invited to participate. Prior to the commencement of the study, detailed information about the research objectives, procedures, potential risks, and benefits was provided to the students. Informed consent was obtained from all participants before data collection began. Ethical approval for the study was obtained from the Ethics Committee of Shiraz University of Medical Sciences with the code IR.SUMS.NUMIMG.REC.1402.108.

After obtaining study approval and the ethics code from Shiraz University of Medical Sciences, the researcher held a meeting with the students in a classroom 1 day before the start of the semester classes, in which she clearly explained the objectives and, after inviting the students to participate, obtained consent forms from the students. All sixth‐semester nursing students who had enrolled in the 1.5 credit theoretical crisis course were included in the study.

#### 2.5.1 Control Group

In the control group, students received conventional face‐to‐face instruction. The instructor delivered the course content during scheduled class sessions using lecture‐based methods, including PowerPoint presentations, verbal explanations, and textbook‐based teaching. Students attended class to receive information directly from the instructor and were expected to take notes and study the materials after class. Classroom time was primarily teacher‐centered with limited opportunities for discussion, group activities, or active participation.

#### 2.5.2 Intervention Group

In the intervention group, in the first session, the students were divided into 9 groups of 5, and a representative was appointed for each group. Then, to familiarize the students with the intervention, the necessary explanations regarding the flipped classroom were provided. Thus, 6 days before class, the PowerPoints and a number of questions were sent to students using the Navid system (an online system within the faculty). Students accessed the educational materials before class through this method, studied them, and reviewed the questions that were posed to them. The instructor posed questions related to the educational content to the students in class and asked them to engage in problem‐solving, discussion, and debate in groups for 45 min. Nursing‐related scenarios were utilized in each session to apply activities to actual clinical experiences and to increase involvement. These comprised triage in mass casualty incidents, communication failure in disasters, and the hospital evacuation for fire. Scenarios prompted students to recognize ethical concerns, to triage care, and to think about the psychological support. Discussion groups facilitated critical thinking, theory application, and self‐reflection regarding nursing's role in disasters. Then, the representative of each group presented the final answers and explained the reasoning behind those answers and a class discussion was held between the groups and the instructor for 30 min (total of 74 min/class). The instructor transitioned from a lecturer to a facilitator, guiding discussions, clarifying ambiguities, and providing real‐time feedback during group activities.

### 2.6 Outcome Measures

Critical thinking and self‐esteem were evaluated via survey at baseline and again postintervention. Retention was measured twice after the intervention to assess learning durability.

Critical thinking skills were measured using the California Critical Thinking Skills Test—Form B (CCTST‐B), which consists of 34 multiple‐choice questions assessing 5 domains: analysis, inference, evaluation, inductive reasoning, and deductive reasoning. The test has been widely validated and demonstrates acceptable reliability (Cronbach's *α* > 0.70). Self‐esteem was measured using the Coopersmith Self‐Esteem Inventory (CSEI), a standardized 50‐item questionnaire rated on a dichotomous (Yes/No) scale, covering general, social, academic, and family dimensions. Learning retention was evaluated using a researcher‐made exam aligned with course objectives, consisting of 40 multiple‐choice questions. The same exam was given as the final test at the end of the semester. To assess retention, an unexpected delayed posttest with similar content and structure was administered 1 month later. Both exams were reviewed by three faculty experts to ensure content validity.

The CCTST‐B is composed of 34 multiple‐choice questions and measures critical thinking ability in terms of 5 ability domains‐: analysis, inference, evaluation, inductive reasoning, and deductive reasoning. The instrument has great validity evidence and acceptable internal consistency for the original one (Cronbach's *α *> 0.70) [22, 23, 24].

Self‐esteem was evaluated with the CSEI, a 35‐item scaled‐response inventory in which participants respond to statements on a 4‐point Likert scale. In Iran, this scale has been psychometrically tested and has a Cronbach's *α* of 0.85 [25, 26].

**Figure 1:**
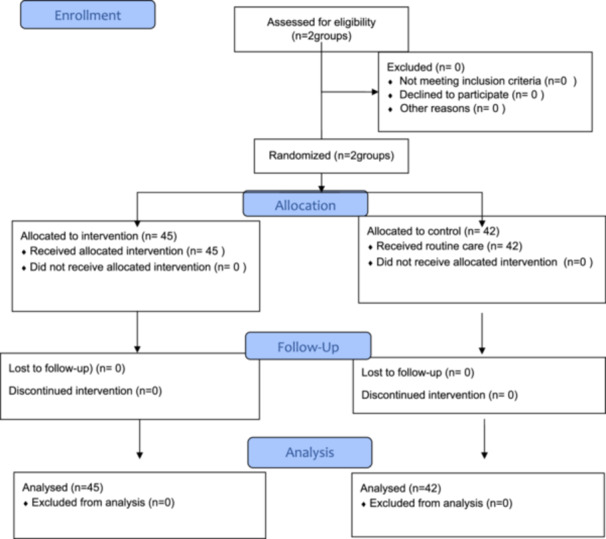
Consort flow chart of the participants.

Retention was measured by a 40‐item multiple‐choice examination developed by a researcher to match the course objectives. The last exam of the semester was identical as the pretest, and an additional surprise delayed posttest with exact content was administered 1 month reassessing the same material. Content validity of both tests was established by nurse educators (a consort diagram was added in Figure 1).

### 2.7 Statistical Analyses

Statistical analysis was performed using SPSS version 21. Descriptive statistics (mean, standard deviation, frequency, and percentage) were used to summarize demographic and baseline characteristics. For inferential analysis, independent *t*‐tests were used to compare between‐group differences, and paired *t*‐tests were applied to evaluate within‐group changes before and after the intervention. *χ* ^2^ tests were used to analyze associations between categorical variables. A significance level of 0.05 and effect sizes (Cohen's *d*) were considered for statistical tests.

### 2.8 Ethical Considerations

Informed consent was obtained from all participants, ensuring confidentiality and anonymity. Participants were also given the right to withdraw from the study at any stage. The study adhered to the 26 ethical principles of research at all stages of the research process and during publication.

## 3 Results

### 3.1 Sample Characteristics

As shown in Table 1, the mean age of participants in the intervention group was 22.46 years (SD = 1.09), while in the control group, it was 22.61 years (SD = 1.24), with no significant difference between the two groups (*p* = 0.547). The average GPA was also similar across groups, with the intervention group averaging 16.26 (SD = 1.11) and the control group averaging 16.19 (SD = 1.15) (*p* = 0.755).

**Table 1:** Demographic characteristics of participants in the intervention and control groups.

Variable		Intervention (*N* = 45)	Control (*N* = 42)	*p*
Quantitative variables^a^, mean ± SD				
Age (year)		22.46 ± 1.09	22.61 ± 1.24	0.547
Grade point average^c^		16.26 ± 1.11	16.19 ± 1.15	0.755
Qualitative variables^b^, *N* (%)				
Gender	Female	25 (55.6)	23 (54.8)	0.556
	Male	20 (44.4)	19 (45.2)	
Job	Unemployed	45 (100)	42 (100)	0.665
	Part‐time	0 (0)	0 (0)	
	Full‐time	0 (0)	0 (0)	

a Two independent sample *T*‐test.

b *χ* ^2^ test.

^c^ GPA.

Sex distribution was comparable between groups. In the intervention group, 25 participants (55.6%) were female and 20 participants (44.4%) were male. In the control group, there were 23 females (54.8%) and 19 males (45.2%) (*p* = 0.556).

**Figure 2:**
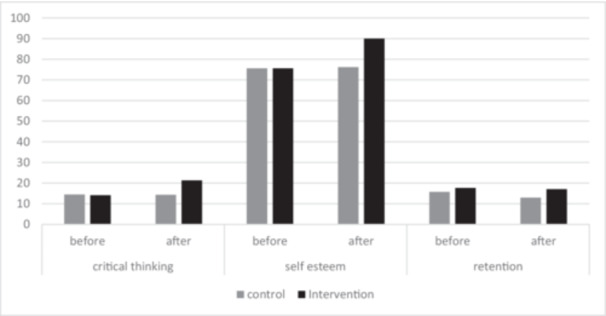
Comparative analysis of critical thinking, self‐esteem, and retention scores between intervention and control groups across time points.

The results of Table 2 showed that critical thinking scores in the intervention group increased significantly from 14.13 ± 1.07 before the intervention to 21.33 ± 1.01 after the intervention (*p* < 0.001, *d* = 1.8), whereas no significant change was observed in the control group (14.45 ± 1.68 before, 14.33 ± 1.47 after; *p* = 0.5). Similarly, self‐esteem scores showed a significant rise in the intervention group, from 75.59 ± 15.49 preintervention to 90.03 ± 9.04 postintervention (*p* < 0.001, *d* = 1.2), while no notable difference occurred in the control group (75.59 ± 15.49 pre, 76.19 ± 13.73 post; *p* = 0.5) (Figure 2).

**Table 2:** Comparative analysis of critical thinking, self‐esteem, and retention scores between intervention and control groups across time points.

Variable	Time	Group		*p*_between_
		Intervention	Control	
Critical thinking	Before	14.13 ± 1.07	14.45 ± 1.68	0.5
	After	21.33 ± 1.01	14.33 ± 1.47	*p* < 0.001
*p*_within_		*p* < 0.001	0.5	
Self‐Esteem	Before	75.59 ± 15.49	75.59 ± 15.49	0.5
	After	90.03 ± 9.04	76.19 ± 13.73	*p* < 0.001
*p*_within_		*p* < 0.001	0.5	
Retention	Immediately^a^	17.66 ± 0.97	15.78 ± 1.1	*p* < 0.001
	1 month later	17.04 ± 1.02	12.97 ± 1.7	*p* < 0.001
*P*_within_		*p* < 0.001	0.5	

a Immediately postintervention.

Retention scores also demonstrated substantial improvement in the intervention group, with scores immediately postintervention (17.66 ± 0.97) and 1 month later (17.04 ± 1.02) both remaining high (*p* < 0.001). In contrast, retention scores in the control group did not significantly improve, with a score of 15.78 ± 1.1 immediately postintervention (*p* < 0.001, *d* = 0.9) and 12.97 ± 1.7 1 month later (*p* = 0.5, *d* = 1.5). Overall, the intervention group exhibited significant enhancements across all three variables compared to the control group, underscoring the intervention's positive impact on critical thinking, self‐esteem, and retention (Figure 2).

## 4 Discussion

This study, which was quasi‐experimental in nature, aimed to determine how nursing students' critical thinking, self‐esteem, and content retention were affected by flipped classroom approach. The findings revealed a statistically significant improvement in the mean scores of critical thinking, self‐esteem, and retention in the intervention group compared to the control group after the intervention (*p* < 0.001). Notably, while the intervention group showed significant gains before and after the implementation, no significant change was observed in the control group (*p* > 0.05).

These results align with previous research showing that flipped classrooms enhance critical thinking, self‐esteem, and information recall among students. For example, Baytiyeh and Naja found that the flipped approach significantly improved critical thinking and problem‐solving among engineering students [27].

Branden's theory suggests that an individual's motivation and self‐confidence for success play a major role in how much time and effort they put into a new circumstance [9]. In explaining the results of this study, it can be stated that in the flipped classroom, elements such as in‐class challenge, preclass study, and group participation affect students' extrinsic and intrinsic motivation and lead to their better performance. Group discussion makes learners more motivated, and its integration with acquired information increases students' cognitive, behavioral, and emotional engagement [28].

Results from a study by Ahadieat that sought to determine how the flipped classroom affected electrical engineering students' learning and satisfaction with the teaching approach were in line with the current study's findings. So that, at the conclusion of the semester, students in the flipped group received higher grades than those in the standard class. They were also more satisfied with this teaching strategy than the students in the control group [29].

In this method, students learn how to improve their learning and use all their mental abilities in different ways. Using critical thinking and problem‐solving skills helps consolidate findings in memory and improves the rate of recall of materials. In a different study, Dehghanzadeh and colleagues demonstrated that the flipped classroom teaching method has a positive effect on nursing students' propensity for critical thinking, stating that the use of innovative approaches has increased students' propensity for critical thinking [30]. Students' poor critical thinking skills are mostly caused by the usage of conventional teaching methods. According to their findings, the educational disparities in promoting critical thinking are the reason why Iranian students exhibited this talent at a lesser level than students in comparable research conducted elsewhere [31].

The positive effects on self‐esteem observed in this study are supported by Branden's theory of self‐efficacy, which aligns with the results of his research on nursing students, indicating that the average self‐esteem score in the flipped classroom had increased compared to the conventional classroom. Ultimately, they concluded that the flipped classroom is more effective than conventional teaching methods if the conditions for implementation are provided [21]. Kim also showed in her study that the flipped classroom has a positive impact on the self‐esteem of nursing students. She states that the use of the flipped classroom approach, compared to the conventional approach, has a positive impact on self‐esteem, which aligns with the results of the present study [32]. According to Branden's theory, which emphasizes mastery experiences, social persuasion, and vicarious learning as key sources of self‐confidence [9]. In this study, the flipped classroom likely provided students with chances to prepare for class (mastery of material) and collaborated (social persuasion using peers' feedback) in the classroom, which increases the students' confidence in their clinical reasoning abilities. Structured and prepared group debates allowed students to witness the problem‐solving processes of peers, which is vicarious learning and builds self‐efficacy.

From the perspective of CLT, the flipped classroom helps reduce extraneous cognitive load by transferring basic content acquisition to preclass activities. This enables more effective use of working memory during in‐class sessions for higher‐order tasks like clinical reasoning and decision‐making. For nursing students, this facilitates a deeper focus on managing complex patient care scenarios in a safe, academic setting. The flipped classroom also aligns with constructivist learning principles. Its student‐centered, active engagement format enables learners to apply knowledge to realistic clinical problems such as triage decisions, disaster response, and communication under pressure through structured discussion and collaboration. This approach strengthens clinical judgment, enhances decision‐making, and bridges the gap between academic theory and real‐world nursing practice [33].

Implications of this study suggest that the use of the flipped classroom approach can improve student engagement, critical thinking skills, and self‐esteem but comes at a cost of additional preparation time and faculty mentorship. Nursing faculty need to develop high‐quality content, use preclass materials, and orient students to the flipped method. The prognostic value of these new approaches with respect to effects on clinical decision‐making and patient care needs to be investigated in a longer follow‐up. Cross specialties and academic levels comparison studies could also contribute to a more nuanced understanding of where flipped classrooms are most effective [34].

### 4.1 Strengths and Weaknesses

The research design involved a full semester of nursing students and the assessment of cognitive (critical thinking, recall) and affective (self‐esteem) outcomes. The study employed real‐time didactics and classroom participation. However, the sample was biased toward one institution, and the potential influence of block randomization (instructor effect) or different group dynamics was not controlled for. Furthermore, the longer‐term effect on clinical performance was not evaluated, and recommendations for future research include studies on workplace outcomes or clinical simulations.

## 5 Final Conclusions

Based on the research conducted in the field of flipped classrooms, it can be stated that teaching using the flipped method can lead to an increase in self‐esteem, critical thinking, and final exam score among nursing students. In addition, the flipped classroom can become a collaborative space with the student's prior preparation. This method, in addition to the mentioned benefits, increases learning motivation and interaction, enhances learning skills, self‐confidence, communication, and self‐regulated learning skills of the student. Therefore, it would seem necessary to have more tangible monitoring of students' performance for self‐learning at the start of the flipped classroom period, particularly in the first few weeks of the semester, in order to help students manage their learning and adjust to the new learning environment. However, since students are used to the lectures and problem‐solving methods that are employed in the majority of the nation's institutions, it is necessary to prepare them for the shift in teaching methods and the implementation of new strategies, such as the flipped classroom.

## Author Contributions

**Mahsa Maryami:** writing – review and editing, resources, writing – original draft, data curation. **Mahboobeh Hosseini Moghaddam:** writing – original draft, writing – review and editing, resources, data curation. **Khatereh Rostami:** writing – original draft, writing – review and editing, resources, data curation. All of the authors approved the draft of the manuscript. All authors have read and approved the final version of the manuscript.

## Ethics Statement

Ethical approval for the study was obtained from the Ethics Committee of Shiraz University of Medical Sciences with the code IR.SUMS.NUMIMG.REC.1402.108. The study adhered to the 26 ethical principles of research at all stages of the research process and during publication. The study adhered to the 26 ethical principles of research at all stages of the research process and during publication.

## Consent

Informed consent was obtained from all participants, ensuring confidentiality and anonymity.

## Conflicts of Interest

The authors declare no conflicts of interest.

## Transparency Statement

The lead author Khatereh Rostami affirms that this manuscript is an honest, accurate, and transparent account of the study being reported; that no important aspects of the study have been omitted; and that any discrepancies from the study as planned (and, if relevant, registered) have been explained.

## Data Availability

The data that support the findings of this study are available from Mahsa Maryami but restrictions apply to the availability of these data, which were used under license for the current study, and so are not publicly available. Data are, however, available from the authors upon reasonable request and with permission of Mahsa Maryami. The authors confirm that the data supporting the findings of this study are available within the article or its Supporting Materials. All authors have full access to all of the data in this study, and take complete responsibility for the integrity of the data and the accuracy of the data analysis.

## References

[1] K. E. Lee , “Effects of Team‐Based Learning on the Core Competencies of Nursing Students: A Quasi‐Experimental Study,” Journal of Nursing Research 26, no. 2 (2018): 88–96, 10.1097/jnr.0000000000000259. 29521887

[2] M. I. Soltanabadi , S. Izadpanah , and E. Namaziandost , “The Effect of Flipped Classroom on Iranian Adolescents: Elementary EFL Learners' Vocabulary Recall and Retention,” Education Research International 2021 (2021): 1–9, 10.1155/2021/3798033.

[3] J. G. Guerrero , S. A. A. Ali , and D. M. Attallah , “The Acquired Critical Thinking Skills, Satisfaction, and Self Confidence of Nursing Students and Staff Nurses Through High‐Fidelity Simulation Experience,” Clinical Simulation in Nursing 64 (2022): 24–30, 10.1016/j.ecns.2021.11.008.

[4] L. Elder and R. Paul , Critical Thinking: Learn the Tools the Best Thinkers Use: Foundation for Critical Thinking (Bloomsbury Publishing, 2020).

[5] Z. Zulhamdi , H. Rahmatan , W. Artika , A. U. T. Pada , and I. Huda , “The Effect of Applying Blended Learning Strategies Flipped Classroom Model on Students' Critical Thinking Skills,” Jurnal Penelitian Pendidikan IPA 8, no. 1 (2022): 86–93, 10.29303/jppipa.v8i1.1186.

[6] M. Lavoie‐Tremblay , L. Sanzone , T. Aubé , C. Bigras , G. Cyr , and G. Primeau , “A University/Healthcare Institution Mentorship Programme: Improving Transition to Practice for Students,” Journal of Nursing Management 28, no. 3 (2020): 586–594, 10.1111/jonm.12960. 31958196

[7] H. Mashalchi , F. Pelarak , S. Mahdavi Kian , T. Mahvar , A. Abdolvand , and M. Habibi Moghadam , “The Effect of Mentorship Program on Self‐Esteem, Anxiety and Learning Clinical Skills of Emergency Medical Students: A Randomized Controlled Trial,” Avicenna Journal of Nursing and Midwifery Care 29, no. 3 (2021): 210–219, 10.30699/ajnmc.29.3.210.

[8] L. Valizadeh , V. Zamanzadeh , R. B. Gargari , A. Ghahramanian , F. J. Tabrizi , and B. Keogh , “Pressure and Protective Factors Influencing Nursing Students' Self‐Esteem: A Content Analysis Study,” Nurse Education Today 36 (2016): 468–472, 10.1016/j.nedt.2015.10.019. 26586259

[9] N. Branden , The Power of Self‐Esteem (Health Communications, Inc., 2021).

[10] K. W. Cho and A. Powers , “Testing Enhances Both Memorization and Conceptual Learning of Categorical Materials,” Journal of Applied Research in Memory and Cognition 8, no. 2 (2019): 166–177, 10.1016/j.jarmac.2019.01.003.

[11] C. S. Ugwuanyi , C. C. Nduji , U. C. Elejere , and N. E. Omeke , “Effect of Flipped Classroom and Think Pair Share Strategy on Achievement and Retention Among Senior Secondary School Physics Students,” International Journal of Sciences: Basic and Applied Research (IJSBAR) 52, no. 2 (2020): 136–148.

[12] C. Torabizadeh , Z. Mahnazrakhshan , and B. Njimehbeygi , “Professional Capability in Nursing,” International Journal of Pharmaceutical Research 11, no. 1 (2019): 556–566, 10.31838/ijpr/2019.11.01.075.

[13] W. M. Attiya and M. B. Shams , “Predicting Student Retention in Higher Education Using Data Mining Techniques: A Literature Review,” in 2023 International Conference on Cyber Management and Engineering (CyMaEn), Bangkok, Thailandd (2023), 171–177, 10.1109/CyMaEn57228.2023.10051056.

[14] M. Förster , A. Maur , C. Weiser , and K. Winkel , “Pre‐Class Video Watching Fosters Achievement and Knowledge Retention in a Flipped Classroom,” Computers & Education 179 (2022): 104399, 10.1016/j.compedu.2021.104399.

[15] E. K. L. Ng , “Flipped Versus Traditional Classroom and Student Achievement and Cognitive Engagement in an Associate Degree Nursing Fundamental Course,” Nurse Education in Practice 68 (2023): 103567, 10.1016/j.nepr.2023.103567. 36758445

[16] J. C.‐Y. Sun and H.‐S. Lin , “Effects of Integrating an Interactive Response System Into Flipped Classroom Instruction on Students' Anti‐Phishing Self‐Efficacy, Collective Efficacy, and Sequential Behavioral Patterns,” Computers & Education 180 (2022): 104430, 10.1016/j.compedu.2022.104430.

[17] A. Ettien and Y. É. J. Touré , “Theoretical Foundations of the Flipped Classroom,” European Journal of Education and Pedagogy 4, no. 6 (December 2023): 53–57, 10.24018/ejedu.2023.4.6.771.

[18] M. S. Ghasemi , G. Ahghar , and D. Taghvaei , “Comparing the Effectiveness of Flipped Teaching and Teaching Metacognitive Strategies in Science Lessons on Students' Self‐Efficacy,” Technology of Education Journal (TEJ) 17, no. 1 (2022): 197–208, 10.22061/tej.2022.9220.2809.

[19] A. Badeleh and E. Izadikhah , “Comparison of Second Grade Female Students' Amount of Learning and Retention of Sciences Lesson Through the Webquest, Mobile and Flipped Training Methods,” New Educational Approaches 14, no. 2 (2019): 21–44, 10.22108/nea.2020.108368.1175.

[20] J. Hill , K. Berlin , J. Choate , L. Cravens‐Brown , L. McKendrick‐Calder , and S. Smith , “Exploring the Emotional Responses of Undergraduate Students to Assessment Feedback: Implications for Instructors,” Teaching & Learning Inquiry 9, no. 1 (2021): 294–316, 10.20343/teachlearninqu.9.1.20.

[21] M.‐K. Cho and M. Y. Kim , “Factors Influencing SDL Readiness and Self‐Esteem in a Clinical Adult Nursing Practicum After Flipped Learning Education: Comparison of the Contact and Untact Models,” International Journal of Environmental Research and Public Health 18, no. 4 (2021): 1521, 10.3390/ijerph18041521. 33562861 PMC7915011

[22] P. A. Facione , N. C. Facione , S. Blohm , and C. Gittens , *The California Critical Thinking Skills Test Manual: Form A and Form B* (CAP, 1992).

[23] H. Khalili and M. Hosseinzadeh , “Investigation of Reliability, Validity and Normality Persian Version of the California Critical Thinking Skills Test; Form B (CCTST),” Journal of Medical Education 3, no. 1 (2003): 29–33, 10.22037/jme.v3i1.871.

[24] C. A. Stone , L. J. Davidson , J. L. Evans , and M. A. Hansen , “Validity Evidence for Using a General Critical Thinking Test to Measure Nursing Students' Critical Thinking,” Holistic Nursing Practice 15, no. 4 (2001): 65–74. 12120497 10.1097/00004650-200107000-00010

[25] S. Coopersmith , *The Antecedents of Self‐Esteem* (Princeton, 1965).

[26] F. Gholami Booreng , B. Mahram , and H. Kareshki , “Construction and Validation of a Scale of Research Anxiety for Students,” Iranian Journal of Psychiatry and Clinical Psychology 23, no. 1 (2017): 78–93, 10.18869/NIRP.IJPCP.23.1.78.

[27] H. Baytiyeh and M. K. Naja , “Students' Perceptions of the Flipped Classroom Model in an Engineering Course: A Case Study,” European Journal of Engineering Education 42 (2017): 1048–1061, 10.1080/03043797.2016.1252905.

[28] M. Eslami , Y. Mahdavinasab , Z. Khoshneshin , and A. Modarressi Saryazdi , “The Effect of Gamification in Flipped Classroom on the Critical Thinking of Female Child Trainer Students in Yazd Technical and Vocational College,” Journal of Educational Psychology Studies 20, no. 51 (November 2023): 18, 10.22111/jeps.2023.44059.5248.

[29] Ahadieat , “The Effect of the Flipped Classroom on the Learning Rate and Satisfaction With the Teaching Method in Electrical Engineering Students of Islamic Azad University, Mehriz Branch,” Iranian Engineering Education Quarterly 20, no. 80 (2019): 51–69, 10.22047/ijee.2019.149317.1580.

[30] S. Dehghanzadeh , F. Jafaraghaie , and A. H. Khordadi , “The Effect of Flipped Classroom on Critical Thinking Disposition in Nursing Students,” Iranian Journal of Medical Education 18 (2018): 39–48, 10.1016/j.nedt.2018.09.027. 30286373

[31] Z. Maleki and M. Rezaee , “Medical Sciences Students' Critical Thinking Skills and the Effect of the University Curriculum: A Literature Review,” *Scientific Journal of Rehabilitation Medicine* 4, no. 4 (2015): 156–165.

[32] J. Kim and N. H. Cha , “Effect of Flipped Learning on the Learning Attitude, Self‐Esteem, Lesson Satisfaction for Nursing Students of the University,” Journal of the Convergence on Culture Technology 8, no. 3 (2022), 10.17703/JCCT.2022.8.3.9.

[33] V. Betihavas , H. Bridgman , R. Kornhaber , and M. Cross , “The Evidence for ‘Flipping Out’: A Systematic Review of the Flipped Classroom in Nursing Education,” Nurse Education Today 38 (March 2016): 15–21, 10.1016/j.nedt.2015.12.010. 26804940

[34] P. Ahmadi , Z. Zarean , and M. Ebrahimpor , “The Effect of the Flipped Learning Method on the Self‐Esteem of Elementary School Students,” Quarterly Journal of Education Studies 9, no. 34 (2023): 35–48, 10.1016/j.edurev.2020.100314.

